# Palliative patients cared for at home by PAMINO-trained and other GPs – health-related quality of life as measured by QLQ-C15-PAL and POS

**DOI:** 10.1186/1472-684X-11-13

**Published:** 2012-08-21

**Authors:** Katja Hermann, Peter Engeser, Joachim Szecsenyi, Antje Miksch

**Affiliations:** 1 Department of General Practice and Health Services Research, University Hospital Heidelberg, Heidelberg, Germany

## Abstract

**Background:**

To maintain patients’ quality of life is one of the major goals in palliative home care provided by general practitioners (GPs). GPs need adequate training to care for palliative patients. The paper seeks to evaluate whether a specific training in Germany (PAMINO) has any improving impact on the care of palliative patients and their health-related quality of life.

**Methods:**

From September 2007 until June 2009, GPs and their palliative care patients with cancer participated in a study to evaluate palliative courses for GPs offered by a regional palliative care initiative (PAMINO). For a period of six months at most or until death, patients were asked monthly to judge their quality of life on the Quality of Life Questionnaire Core 15 Palliative (QLQ-C15-PAL) of the European Organization for Research and Treatment of Cancer (EORTC) and on the Palliative Care Outcome Scale (POS). The ‘Overall quality of life’ scale of the QLQ-C15-PAL takes values between 0 and 100 with higher values indicating a higher quality of life. The POS sum scale takes values between 0 and 40 with higher values indicating worse care outcomes. Patients cared for by PAMINO-trained GPs and patients cared for by other GPs (control group) are compared using t-tests for differences in group means.

**Results:**

One hundred patients participated in the study; 96 patients filled out the questionnaires at least once. On the QLQ-C15-PAL, mean quality of life of the patient groups of PAMINO-trained and other GPs were 37.7 (SD = 25.5) and 39.4 (SD = 26.3) (p = .76), respectively. On the POS, respective mean values of 13.6 (SD = 5.8) and 12.0 (SD = 6.5) (p = .26) were given. Patients cared for by a PAMINO-trained GP did not report better quality of life and care outcomes than patients cared for by other general practitioners.

**Conclusions:**

Patients cared for by PAMINO-trained and other GPs in our study did not report differences in quality of life. Quality of life and care outcomes of all patients were better than of palliative patients in institutional or specialized care, emphasizing the ability of GPs to provide adequate care for these vulnerable patients. However, conclusions need to be drawn cautiously since the study had a small sample size.

**Trial registration:**

Current Controlled Trials ISRCTN78021852

## Background

To maintain patients’ quality of life (QoL) is one of the major goals in palliative care.

For patients cared for at home, general practitioners (GPs) play an important role in providing the necessary medical support, since they are often the first and major contact person for patients and caregivers. They know private and familial circumstances and are long-term confidants of the patients. They often accompany patients during the whole disease trajectory. For a majority of patients, primary palliative care – as provided by GPs and home care nursing services – is sufficient, although adequate training should be given to care providers [1-4].

In Germany, palliative care is obligatory during the medical curriculum only since 2009. Medical students hardly get into contact with palliative care issues. However, once physicians receive a board certification as a specialist, they might further train to get an additional qualification in palliative medicine. This additional qualification is not a prerequisite for caring for palliative patients.

In 2003, a regional initiative was founded in the federal state of Baden-Wuerttemberg to improve outpatient palliative care (Palliativmedizinische Initiative Nordbaden, PAMINO) [5,6]. Within this initiative, a special focus is laid on general practitioners: vocational training courses required for the additional qualification were developed and are offered by GPs for GPs. Additionally, participating GPs organize themselves in a network with regular meetings to provide collegial feedback and support [6].

This study sought to evaluate if palliative patients of GPs trained in palliative care have a better health-related QoL.

## Methods

From September 2007 until June 2009, GPs and their palliative care patients participated in a study to evaluate palliative courses for GPs offered by a regional palliative care initiative (PAMINO). For a period of six months or until death (if the patients died within the six-month observation period), patients were asked monthly to judge their quality of life on the Quality of Life Questionnaire Core 15 Palliative (QLQ-C15-PAL) of the European Organization for Research and Treatment of Cancer (EORTC) [7] and on the Palliative Care Outcome Scale (POS) [8]. Within the study, no intervention or instruction regarding care was given, but GPs carried out their normal duties. The study was conducted in accordance with the Helsinki Declaration. The study protocol was approved by the ethics committee of the Medical Faculty Heidelberg (S-043/2007). The study was registered (ISRCTN78021852) and the study protocol was published [9].

### Participants

To be eligible for the study, GPs had to take care of palliative patients. The group of PAMINO-trained GPs (PG) had to have completed at least the 40-hours basic training course in palliative care. There were no further restrictions on GPs in the control group (CG); they should not have attended another palliative care training, but this was not an explicit exclusion criterion. GPs volunteered to participate in the study. All PAMINO-trained GPs and a random sample of other GPs from the same region were invited to include patients in the study.

Patients were eligible for inclusion in the study if they fulfilled the following criteria: (a) being in a palliative situation with cancer, where the GP would not be surprised if they died within 6 months, and having no other disease with a lower life expectancy, (b) adult (at least 18 years of age), (c) sufficient command of German to understand the study information and the questionnaires and (d) outpatient care by a GP who participated in the study as well. Patients and GPs had to give their informed and written consent to participate.

### Data collection

Participating GPs informed eligible patients in their practice about the study. Patients were only included if they consented to participate. After inclusion in the study, GPs once a month gave patients a questionnaire containing the QLQ-C15-PAL and the POS. Patients sent the questionnaires to the study centre in postage-paid return envelopes immediately after they filled them out. For study purposes (follow-up), patients were given a pseudonym number printed on the questionnaires to ensure confidentiality. The study centre was not able to identify patients personally; GPs were not informed of patients’ individual answers.

The Quality of Life Questionnaire Core 15 Palliative (QLQ-C15-PAL) [7] was developed as a core instrument to measure QoL especially in cancer patients in palliative care. It consists of 15 questions which are transformed into two function scales (‘Physical Functioning’, ‘Emotional Functioning’), seven symptom scales (‘Fatigue’, ‘Nausea/Vomiting’, ‘Pain’, ‘Dyspnoea’, ‘Insomnia’, ‘Appetite loss’, ‘Constipation’) and an ‘Overall quality of life’ scale. Patients should answer the questions according to their experiences during the previous week. Responses to 14 questions are given on a four-point Likert scale with 1 ‘Not at all’, 2 ‘A little’, 3 ‘Quite a bit’, and 4 ‘Very much’, the question to overall QoL allows answers between 1 ‘Very poor’ and 7 ‘Excellent’. The QoL, function and symptom scales take values between 0 and 100 with higher values indicating a higher QoL, higher functioning and higher symptom burden, respectively.

The Palliative Care Outcome Scale (POS) [8] is used to measure outcome in palliative care. It consists of 12 questions covering the main components of palliative care. Eight questions have a 5-point Likert-scale response from 0 (not at all) to 4 (overwhelming), two questions have 3 answer options (0-2-4), one question (main problems of the previous 3 days) is answered in free text and the last question asks patients if they needed help with filling out the questionnaire (0 – no, 1 – help from family or friend, 2 – help from staff). Patients were asked to answer the questions according to their experiences during the previous 3 days. Although it is not a unidimensional scale [10], a sum score was used as well to describe outcome and to compare groups next to single items [8,11] with higher scores indicating more severe problems.

GPs recorded patients’ medical problems as well, including the performance status (PS) of the Eastern Cooperative Oncology Group (ECOG) [12]. The ECOG PS takes values between 0 and 4 (0 – fully active, able to carry on all pre-disease performance without restriction, 1 – restricted in physically strenuous activity but ambulatory and able to carry out work of a light or sedentary nature, 2 – ambulatory and capable of all selfcare but unable to carry out any work activities, up and about more than 50 % of waking hours, 3 – capable of only limited selfcare, confined to bed or chair more than 50 % of waking hours, 4 – completely disabled, cannot carry on any selfcare, totally confined to bed or chair).

### Data analysis

Data from patients on QLQ-C15-PAL as well as the POS item and sum scores are described as means (M) with standard deviation (SD). The most recent data are considered, i.e. the last available assessment from the patients before either their death or the end of the 6-month observation period. Since both questionnaires should measure closely related constructs, ‘Overall quality of life’ from the QLQ-C15-PAL and the POS sum score are correlated using Pearson’s correlation coefficient.

Patients cared for by PAMINO-trained GPs (PG) and patients cared for by other GPs (CG) are compared using chi-square test for frequencies and t-tests for differences in group means. To confirm the results and to control for cluster effects in the practices as well as for possible influences of patient and GP characteristics, we additionally conducted regression models. For all tests, p < .05 is considered to be statistically significant. All statistical analyses are conducted using SPSS 15.0 (SPSS Inc., Chicago, Ill.) and SAS 9.2 (SAS Institute Inc., Cary, NC).

For sample size calculation, we assumed that every GP cared for 4 eligible patients who were willing to participate in the study. To show a minimum mean difference of 2.0 points in the sum score of the POS, with an assumed standard deviation of 0.6, power set to 80 %, and controlling for cluster effects, we needed to include 360 patients from 90 practices.

## Results

### GP and patient sample

**Figure 1:**
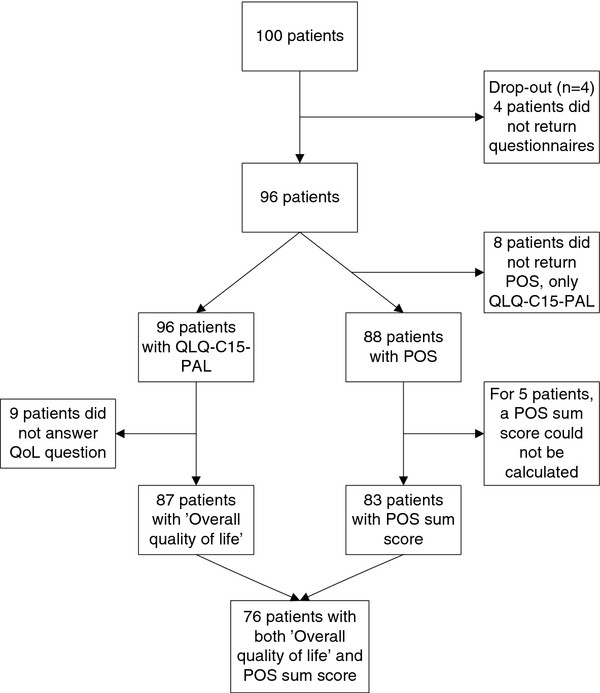
Flowchart of study participants and available data.

There were 100 patients cared for by 45 GPs participating in the study. Four patients did not fill out any questionnaire in the study period; those patients were excluded from further analysis (Figure 1). Sixty-two patients were cared for by 27 PAMINO-trained GPs; 34 patients belonged to 18 other practices. There was one GP with 3 patients in the control group who had a palliative care training other than PAMINO. GPs in both groups did not differ in respect to their demographic background and their years of experience (Table 1). Patient characteristics are shown in Table 2. Patients were between 39 and 91 years old (M = 69; SD = 11.6); 38.5 % were female. The most common diagnoses were lung (n = 13), colon (n = 12) or breast (n = 11) cancer, with 74 patients having at least one comorbidity (e.g. hypertension, diabetes). In the patient group cared for by PAMINO-trained GPs, 56 % of the patients had an ECOG PS of 3 or 4. In the control group of patients cared for by other GPs 49 % of the patients had an ECOG PS of 3 or 4. About 40 % of patients in both groups have had a hospital consultation within the month prior to the study assessment; 7 patients were in contact with palliative care services (including physician, nursing, palliative care unit, and hospice). There were no statistically significant differences between patient groups.

**Table 1:** Sample characteristics of GPs in PAMINO (PG) and control group (CG)

	**PG (n = 27)**	**CG (n = 18)**
Women (%)	9 (33.3)	3 (16.7)
Men (%)	18 (66.7)	15 (83.3)
**Age**; mean yrs (SD)	53.5 (7.3)	54.5 (8.2)
**GP practice experience**; mean yrs (SD)	19.1 (8.1)	18.3 (7.4)
single-handed practice (%)	13 (48.1)	8 (44.4)
other (%)	14 (51.9)	10 (55.6)
**Practice location** (%)		
urban	5 (18.5)	5 (27.8)
suburban	16 (59.3)	9 (50.0)
rural	6 (22.2)	4 (22.2)
**No. of patients per quarter year** (%)		
up to 1000 patients	7 (25.9)	4 (22.2)
1001 to 1500 patients	9 (33.3)	10 (55.6)
more than 1500 patients	11 (40.7)	4 (22.2)
**Palliative care patients per year** (%)		
0-2	-	3 (16.7)
3-5	11 (40.7)	4 (22.2)
6-9	8 (29.6)	5 (27.8)
10 and more	8 (29.6)	6 (33.3)
**Distance to the next palliative care unit**; median km (min-max)	12.5 (1–60)	10 (0.5-80)

**Table 2:** Patient characteristics of palliative patients

	**all (n = 96)**	**PG (n = 62)**	**CG (n = 34)**
**female; n (%)**	37 (38.5)	25 (40.3)	12 (35.3)
**male; n (%)**	59 (61.5)	37 (59.7)	22 (64.7)
**age; M (SD) years**	68.6 (11.6)	69.7 (11.2)	66.7 (12.2)
**living…; n (%)**			
** … alone**	19 (19.8)	14 (22.6)	5 (14.7)
** … with relatives**	72 (75.0)	45 (72.6)	27 (79.4)
** … in day care**	3 (3.1)	2 (3.2)	1 (2.9)
**main cancer diagnosis site; n (%)**			
** lung**	13 (13.5)	9 (14.5)	4 (11.8)
** colon**	12 (12.5)	9 (14.5)	3 (8.8)
** breast**	11 (11.5)	7 (11.3)	4 (11.8)
** stomach**	8 (8.3)	4 (6.5)	4 (11.8)
** prostate**	7 (7.3)	4 (6.5)	3 (8.8)
** other**	45 (46.9)	29 (46.7)	16 (47.1)
**ECOG performance status; n (%)**			
** 0 (fully active)**	9 (9.4)	7 (11.3)	2 (5.9)
** 1 (restricted)**	13 (13.5)	10 (16.1)	3 (8.8)
** 2 (ambulatory)**	21 (21.9)	9 (14.5)	12 (35.3)
** 3 (limited ability)**	16 (16.7)	9 (14.5)	7 (20.6)
** 4 (disabled)**	33 (34.4)	24 (38.7)	9 (26.5)
** missing**	4 (4.2)	3 (4.8)	1 (2.9)

PG patient group cared for by PAMINO-trained general practitioners, CG control group patients cared for by other general practitioners, M mean, SD standard deviation, ECOG Eastern Cooperative Oncology Group.

The QLQ-C15-PAL and the POS are both self-administered questionnaires measuring quality of life. More than half of the patients (PG: 52 %, CG: 63 %, p = .33) needed help from either family/friends or staff to fill out the questionnaires.

### ‘Overall quality of life’ and POS sum score

Patients reported a mean quality of life on the QLQ-C15-PAL of 38.1 (SD = 25.7, n = 87) and on the POS of 13.0 (SD = 6.1, n = 83). Of 76 patients, both questionnaires were available. ‘Overall quality of life’ (QLQ-C15-PAL) and POS sum score correlated highly (r = −.59, p < .01).

On the QLQ-C15-PAL, mean QoL of the patient groups of PAMINO-trained and other GPs were 37.7 (SD = 25.5, n = 54) and 39.4 (SD = 26.3, n = 33) (p = .76), respectively. On the POS, respective mean values of 13.6 (SD = 5.8, n = 51) and 12.0 (SD = 6.5, n = 32) (p = .26) were given. Patients cared for by a PAMINO-trained GP did not report better QoL and care outcomes than patients cared for by another GP.

The results of the univariate analyses were confirmed in regression models using practice as cluster variable and group, ECOG PS, gender and age of the patient, and experience of the GP as independent variables. Due to missing values, the models were analyzed with n = 81 and n = 78 for ‘Overall quality of life” and the POS sum score, respectively. Only the ECOG PS significantly influenced the two scales: Compared to patients with a ECOG PS of 4, patients with a ECOG PS of 0, 1 or 2 had a higher ‘Overall quality of life’, and patients with a ECOG PS of 0 or 1 had a lower POS sum score.

### QLQ-C15-PAL function and symptom scales

On the function scales, patients in both groups reported a higher emotional functioning (M = 46.9, SD = 34.4, n = 95) than physical functioning (M = 30.1, SD = 34.5, n = 92). Additionally, physical functioning was skewed towards the lower end of the scale (median = 13.3). The most prevalent symptoms were fatigue (M = 74.4, SD = 30.1, n = 94), appetite loss (M = 55.1, SD = 40.3, n = 95) and pain (M = 51.1, SD = 36.2, n = 95). Patients in both groups did not differ in their perception of function and symptoms (Table 3).

**Table 3:** Results of the QLQ-C15-PAL (Overall quality of life, Function and Symptom scales)

	**PG**		**CG**		
	**n**	**M (SD)**	**n**	**M (SD)**	***p***
**QLQ-C15-PAL**^**a**^					
**Overall quality of life** (higher values indicating a higher quality of life)	54	37.7 (25.5)	33	39.4 (26.3)	.76
**Function scales** (higher values indicating better functioning)					
Physical functioning	59	30.5 (34.6)	33	29.3 (34.6)	.87
Emotional functioning	61	45.6 (31.8)	34	49.3 (39.2)	.65
**Symptom scales** (higher values indicating greater presence)					
Dyspnoea	60	42.8 (35.3)	33	42.4 (38.4)	.96
Pain	61	51.4 (36.4)	34	50.5 (36.4)	.91
Insomnia	61	44.8 (35.4)	34	49.0 (36.0)	.58
Fatigue	60	75.9 (29.4)	34	71.6 (31.6)	.50
Appetite loss	61	51.4 (40.2)	34	61.8 (40.3)	.23
Nausea/Vomiting	60	23.1 (32.2)	34	26.0 (28.5)	.66
Constipation	60	33.3 (34.2)	34	32.4 (34.3)	.98

^a^ Scale scores ranging from 0 to 100; PG patient group cared for by PAMINO-trained general practitioners, CG control group patients cared for by other general practitioners, M mean, SD standard deviation.

### POS item scores

On the POS items, patients in both groups most often reported an ‘anxious family’ (M = 2.4, SD = 1.4, n = 88) and not ‘feeling good’ (M = 1.9, SD = 1.3, n = 87). There were less problems with ‘waste of time’ (M = 0.3, SD = 0.7, n = 88) and ‘information given’ (M = 0.5, SD = 1.1, n = 87), where more than 80 % of the patients did not report any problems at all. Patients in the two groups did not differ significantly in their perception of the various aspects of care outcomes (Table 4).

**Table 4:** Results of the POS (sum and item scores)

	**PG**		**CG**		
	**n**	**M (SD)**	**n**	**M (SD)**	***p***
**POS** (higher scores indicating worse care outcomes)					
**Sum score**^**a**^	51	13.6 (5.8)	32	12.0 (6.5)	.26
**Items**^**b**^					
Pain	55	1.8 (1.3)	32	1.4 (1.2)	.23
Other symptoms	56	1.8 (1.1)	32	1.6 (1.0)	.35
Feeling anxious	55	1.9 (1.1)	32	1.7 (1.2)	.39
Family anxious	56	2.5 (1.4)	32	2.3 (1.4)	.56
Information given	55	0.5 (1.2)	32	0.3 (0.7)	.30
Share feeling	56	0.6 (0.9)	32	0.5 (0.9)	.71
Life worthwhile	54	2.0 (1.4)	32	1.6 (1.4)	.16
Felt good	55	2.1 (1.3)	32	1.6 (1.3)	.15
Waste of time	56	0.3 (0.7)	32	0.3 (0.7)	.69
Practical problems	53	0.6 (1.1)	32	0.6 (1.1)	.81

^a^ Scale scores ranging from 0 to 40; ^b^ Item scale scores ranging from 0 to 4; PG patient group cared for by PAMINO-trained general practitioners, CG control group patients cared for by other general practitioners, M mean, SD standard deviation.

## Discussion

This study evaluated if there are differences within the health-related QoL of patients cared for by GPs who participated in a palliative training course offered by GPs (PAMINO) compared to patients of other GPs. In our study sample, patients did not report any differences in their QoL and care as measured by QLQ-C15-PAL and POS. The study suggests that PAMINO training makes no noticeable difference to the quality of care for patients between comparable groups of GPs.

We tried to include as many GPs and patients as possible, but did not reach our targeted sample size. GPs either did not care for enough eligible patients or did not participate due to time constraints. There were enough practices participating in the study (n = 90), but only half of them included patients. Mostly, there were less eligible patients in the practices than expected: there were not as many cancer patients as we assumed for our sample size calculation. Therefore, this study has the character of a pilot study and conclusions need to be drawn cautiously. Although our study is underpowered, it nevertheless describes the quality of life in palliative patients cared for by GPs.

Patients considered their QoL to be moderately high. Not surprisingly, QoL was much lower than in the general German population [13], but higher than in comparable palliative care populations [14].

Additionally, GPs in general delivered high-quality care in the patients’ view. Compared to patients cared for in nursing homes [11], they reported better care outcomes. The patients of the German POS validation study [8], who were mostly cared for in palliative care units in hospital, also reported worse care outcomes than our study population.

As was to be expected, both measures correlated highly showing the high interdependence of care outcomes and health-related quality of life as perceived by patients.

Although our study failed to reveal statistical significant differences within the QoL of patients, it does not mean that the initiative had no impact at all. Unlike non-participating doctors, GPs participating in this voluntary training might gain valuable knowledge and skills in caring for palliative patients, which are of increasing importance in the future. Furthermore, we did not evaluate the training from the GPs’ point of view, for which a longitudinal design with focussing on GPs perceptions and attitudes (asking GPs before and after the training) would have been more appropriate. Furthermore, our study sample mostly consisted of middle-aged GPs with long years of experience. The way they care for patients was perhaps not influenced by training but by learning on the job. These issues should be taken into consideration within further research.

GPs had to include eligible patients from their practice in the study. Although there were inclusion criteria, the recruitment of patients was prone to a selection bias, since GPs decided whom they thought eligible. Patients had to be progressed enough in their cancer trajectory and still be able to participate and to fill out the questionnaires. Those closer to the end of life were probably less often approached for study purposes leading to a generally healthier patient sample. Nonetheless, included patients could be followed-up during the study period up to the point of disease progression. The fact, that more than half of the patients needed help in filling-out the questionnaires at the last study assessment, emphasizes their needs and more severe condition.

A major limitation of the study is the choice of the control group. GPs participating in this study were interested in palliative care, independent of whether they did attend further training or not. So, the GPs in the control group were equally eager to deliver high-quality care and to help patients maintaining a high quality of life. But, since GPs not interested in palliative care tend to let other medical professionals (specialists, home care services, hospitals) take care of the patients, a control group providing mere basic care is probably hard to find.

The study was an observational study without further intervention. Still, there might have occurred an observation bias, since patients and GPs alike were made alert to specific aspects of care that they were asked about in the questionnaires and documentations. An influence of the study on the care delivered, and thus on patients’ quality of life, cannot be ruled out.

We might have conducted a study with the training as intervention and pre-post assessments. Such a before and after study would probably not be able to detect the effect of the training either. Patients in a palliative care situation naturally and unpredictably change over (sometimes a very short) time. It is very difficult, for all health professionals involved, to predict the illness trajectory of a single patient. For a study, we need to find the balance between getting important results and too much burden on patients and caregivers.

## Conclusions

Although it is frequently argued that GPs need further training to provide adequate care for palliative patients in home care, patients cared for by PAMINO-trained and other GPs in our study did not report differences in quality of life. However, these results cannot be generalised due to the small sample size. QoL and care outcomes of all patients were better than of palliative patients in institutional or specialized care, emphasizing the ability of GPs to provide adequate care for these vulnerable patients.

## Competing interests

The authors declare that they have no competing interests.

## Authors’ contributions

AM and JS designed the study. KH and PE conducted the study. KH performed the statistical analyses and drafted the manuscript. All authors contributed substantially to the manuscript and approved its final version.

## Pre-publication history

The pre-publication history for this paper can be accessed here:

http://www.biomedcentral.com/1472-684X/11/13/prepub
